# Dry Eyes, Ocular Lubricants, and Use of Systemic Medications Known or Suspected to Cause Dry Eyes in Residents of Aged Care Services

**DOI:** 10.3390/ijerph17155349

**Published:** 2020-07-24

**Authors:** Muhamad Aljeaidi, Claire Keen, J. Simon Bell, Tina Cooper, Leonie Robson, Edwin C. K. Tan

**Affiliations:** 1 Centre for Medicine Use and Safety, Faculty of Pharmacy and Pharmaceutical Sciences, Monash University, 381 Royal Parade, Parkville, VIC 3052, Australia; muhamad.aljeaidi@gmail.com (M.A.); claireskeen@gmail.com (C.K.); simon.bell2@monash.edu (J.S.B.); 2 Resthaven Incorporated, Adelaide, 6 Bartley Cres, Wayville, SA 5034, Australia; tina.cooper@resthaven.asn.au (T.C.); leonie.robson@resthaven.asn.au (L.R.); 3 School of Pharmacy, Faculty of Medicine and Health, The University of Sydney, Camperdown, NSW 2006, Sydney, Australia; 4 Aging Research Center, Department of Neurobiology, Care Sciences and Society, Karolinska Institutet and Stockholm University, Solnavägen 1, 171 77 Solna, Stockholm, Sweden; 5 Stress Research Institute, Department of Psychology, Stockholm University, Frescati hagväg 16, 104 05 Stockholm, Sweden

**Keywords:** dry eye syndromes, drug side effects, aged, dementia, frailty, long-term care

## Abstract

Ocular issues are common, burdensome, and under-researched among residents of aged care services. This study aims to investigate the prevalence of dry eyes or use of ocular lubricants among residents, and the possible association with systemic medications known or suspected to cause dry eyes. A cross-sectional study of 383 residents of six aged care services in South Australia was conducted. Data were extracted from participants’ medical histories, medication charts, and validated assessments. The main exposure was systemic medications known to cause, contribute to, or aggravate dry eyes. The primary outcome was documented dry eyes or regular administration of ocular lubricants. Logistic regression was used to estimate odds ratios (ORs) and 95% confidence intervals (CIs) for the association between systemic medications and dry eyes/use of ocular lubricants. Dry eyes were documented for 53 (13.8%) residents and 98 (25.6%) residents were administered ocular lubricants. Overall, 116 (30.3%) residents had documented dry eyes/used ocular lubricants. Of these, half (*n* = 58) were taking a medication known to cause, contribute to, or aggravate dry eyes. Taking one or more medications listed as known to cause dry eyes was associated with having dry eyes/use of ocular lubricants (OR 1.83, 95% CI 1.15–2.94). In sub-analyses, no individual medication was associated with dry eyes/use of ocular lubricants. Dry eyes and use of ocular lubricants are common in residential aged care. Our hypothesis generating findings suggest the need for further research into the clinical significance of systemic medications as a possible cause of dry eyes.

## 1. Introduction 

Ocular issues are common, burdensome, and under-researched among residents of aged care services (ACSs) [1,2]. Studies have reported that the prevalence of visual complications in residents of ACSs is generally higher than older adults living in the community [3,4]. While ocular complications can impact the life of all older adults, those with dementia and frailty are particularly vulnerable. Due to cognitive and functional impairment, visual dysfunction may further contribute to or exacerbate eye conditions [2,5]. One of the issues that may lead to ocular and visual complications is dry eyes [6].

Dry eye syndrome is a complex disease of the eye characterized by the lack of a stable tear film with symptoms that vary from a mild gritty sensation to severe discomfort that affects the daily living of the individual, and can be accompanied with abnormality of the ocular surface [7]. Dry eye syndrome can impact the quality of life of the individual as it causes dryness, grittiness, visual discomfort, and ocular complications. This increases both the health and economic burden on the individual, caregivers and society [8].

The prevalence of dry eyes is associated with age, with older people more likely to experience dry eye symptoms [9]. It is estimated worldwide prevalence of symptomatic dry eyes in people aged 40 years and older ranges from 20 to 50% [9]. Other risk factors include hazardous environments, inflammatory and other systemic conditions, ophthalmic surgery, and medication use [10]. Medication classes such as antihistamines, anticholinergics, antidepressants, and anxiolytics have been reported to be associated with dry eyes [10]. However, evidence for these associations is generally weak and there is a lack of data on the possible association among residents of ACSs. The Tear Film and Ocular Surface Society (TFOS) Dry Eye Workshop (DEWS) generated a list of medications that are “known or suspected to cause, contribute, or aggravate dry eyes” [10]. The possible mechanisms by which medications may contribute include decreasing tear production, altering tear stability leading to increased evaporation, and inducing inflammatory changes on secretory glands [10,11]. 

Residents of ACSs are generally older and experience higher rates of multimorbidity and polypharmacy than adults living in the community [12]. This may mean this population is susceptible to dry eyes. However, there is a lack of literature in this vulnerable population, including in those with dementia and frailty. Thus, the aim of this study was to investigate the prevalence of dry eyes in residents of ACSs and the possible association between systemic medication use, dementia, frailty, and dry eyes. 

## 2. Materials and Methods 

### 2.1. Sample and Data Source

This was a secondary analysis of cross-sectional data for 383 long-term residents of six ACSs in South Australia in 2014. The included participants were similar to all residents of the ACSs from which they were sampled in terms of age (87.5 years (standard deviation [SD] 6.2) vs. 87.3 years (SD 6.4), *p* = 0.66), sex (77.5% female vs. 78.5% female, *p* = 0.90), and dementia diagnosis (44.1% vs. 46.8%, *p* = 0.72). Collection of data was performed by three experienced and trained study nurses. A detailed description of the study design has been published previously [13]. In short, medical diagnoses were extracted from medical histories of all participants and information on medications were extracted from participants’ medication charts. Other clinical data were obtained using validated scales suitable for use among people with and without dementia. 

### 2.2. Medication Exposure

The main exposure was systemic medications listed as being suspected or known to cause, contribute to or aggravate dry eyes. This list was outlined in the TFOS DEWS II iatrogenic report. The report includes 118 suspected medications of which 40 were considered as being known to be associated with dry eyes. A detailed list is reported in Table S1. All medications charted as regular or as-required were included and were categorized using the Anatomical Therapeutic Chemical (ATC) classification system [14]. 

### 2.3. Outcome

The primary outcome was a diagnosis of dry eyes recorded in the medical record or the regular administration of lubricating eye drops/ointments (ATC codes: S01XA20 and S01KA02) at least daily within the previous week. Regular administration of lubricating eye drops/ointments was investigated in addition to documented dry eyes to account for possible under-documentation of dry eyes in the medical record. Eye drops/ointments could be either administered by ACS staff or self-administered by the resident. However, most residents of Australian ACSs do not self-administer medications and administration is typically performed by registered nurses, enrolled nurses, or personal care assistants.

### 2.4. Covariates

Covariates included age, sex, history of ophthalmic conditions, the Charlson comorbidity index (CCI), systemic conditions associated with dry eyes, dementia severity, and frailty. The Charlson comorbidity index was used as a measure of comorbidity and disease severity. It is a method of weighting comorbidities to give a single comorbidity score for each individual based on their relative risk of mortality [15]. Presence or history of an ophthalmic condition can influence the prevalence of dry eyes [16,17]. Ophthalmic conditions included presence or history of glaucoma, use of glaucoma eye drops, cataracts, macular degeneration, and other ophthalmic conditions, including surgery. Systemic conditions that are reported or suspected to cause or worsen dry eyes included Sjögren’s syndrome, diabetes, arthritis, osteoporosis, asthma, Parkinson disease, and thyroid/hormonal dysfunction [9,18]. The Dementia Severity Rating Scale (DSRS) was used to measure dementia severity in all residents both with and without a documented dementia diagnosis. A DSRS score of >18 is considered moderate to severe severity [19]. The FRAIL-NH screening tool was used to assess frailty [20]. The scale was constructed using clinical data and includes seven items: fatigue, resistance, ambulation, incontinence, loss of weight, nutrition, and dressing. 

### 2.5. Statistical Analysis

Descriptive statistics were used to compare participants’ baseline characteristics according to documentation of dry eyes or administration of ocular lubricants. Binary logistic regression was used to estimate odd ratios (ORs) and 95% confidence intervals (CIs) for the association between systemic medications known or suspected to cause dry eyes, dementia and frailty with documented dry eyes or use of ocular lubricants. Model 1 was adjusted for age and sex, while Model 2 was additionally adjusted for history of ophthalmic conditions, CCI, number of systemic conditions, dementia severity, frailty, and medication exposure where appropriate. The use of glaucoma drops was excluded from the analysis to avoid potential multicollinearity with glaucoma diagnosis. Two sets of sub-analyses were also performed. Firstly, each of the main medication classes listed as being known to cause, contribute to, or aggravate dry eyes were investigated for their association with dry eyes or administration of ocular lubricants. Secondly, the main analyses were repeated when the primary outcome was limited to those residents who had a diagnosis of dry eyes documented in the medical record. All analyses were performed in SAS version 9.4 (SAS Institute, Inc., Cary, NC, USA).

### 2.6. Ethical Approval

Ethical approval was obtained by The Royal Australian College of General Practitioners National Research and Evaluation Ethics Committee and the Monash University Health Research Ethics Committee. Written informed consent was obtained from all participants prior to inclusion. Where residents were unable to provide informed consent, this was obtained from a guardian, next of kin, or significant other.

## 3. Results

A total of 383 participants were included in this study. Dry eyes was present in 30.3% (*n* = 116) of residents; this included 53 residents with a documented diagnosis of dry eyes in the medical record, and 98 residents regularly administered lubricating eye drops/ointments. Of those with dry eyes or using ocular lubricants, 78.5% (*n* = 91) were aged 85 years or older and the same proportion were female (Table 1). Those with dry eyes or using ocular lubricants had moderate frailty (mean (SD) FRAIL-NH 5.10 [4.4]), and 43% (*n* = 49) had moderate-to-severe dementia severity. All residents with dry eyes or using ocular lubricants (*n* = 116) were taking one or more medications listed by the TFOS as being suspected to cause, contribute to, or aggravate dry eyes. The most commonly used of these medications were vitamins (78%), atenolol (16%) and mirtazapine (13%). Half (*n* = 58) of the residents with dry eyes or using ocular lubricants took one or more medications listed as being known to cause, contribute to, or aggravate dry eyes. The most commonly used of these medications were aspirin (34%), diazepam (4%), and propranolol (2%). There were no statistically significant associations between any individual class of medication and dry eyes or using ocular lubricants. 

Table 2 shows the age and sex-adjusted and fully-adjusted odds ratios for having dry eyes or using ocular lubricants. In the second, fully adjusted model, compared to non-users, taking one or more medications listed as being known to cause, contribute to, or aggravate dry eyes was associated with having dry eyes or using ocular lubricants (OR = 1.83 (95%CI 1.15 to 2.94), *p* = 0.01). Similarly, frailty was associated with dry eyes or using ocular lubricants (OR = 1.11 (95 CI 1.02 to 1.19), *p* = 0.01). Dementia was inversely associated with dry eyes or ocular lubricants use (OR = 0.47 (95%CI 0.25 to 0.88), *p* = 0.02). 

When the analyses was limited to having documented dry eyes only, taking one or more medications listed as being known to cause, contribute to, or aggravate dry eyes remained significantly associated with having documented dry eyes (OR = 2.51 (95%CI 1.33 to 4.73), *p* < 0.01) (Table S2). However, frailty and dementia severity were no longer associated with documented dry eyes.

## 4. Discussion

To our knowledge, this is the first study to specifically investigate the prevalence of dry eyes and use of ocular lubricants among residents of ACSs. Our study found that a third of residents had either dry eyes or use of ocular lubricants. The use of one or more medications listed as being known to cause, contribute to, or aggravate dry eyes was associated with higher odds of documented dry eyes or use of ocular lubricants. Residents with greater frailty had higher odds of dry eyes or use of ocular lubricants while those with greater dementia severity had lower odds. 

Few studies have investigated the prevalence of dry eyes in the ACS setting. An earlier study by Handelman et al. investigated the association between hyposalivatory medications, dry mouth, and salivary flow [21]. Their study reported a 30% prevalence of perceived eye dryness in residents of ACSs who took medications known to cause hyposalivation [21]. Previous studies have investigated the prevalence of dry eyes in the general older population. A review of eight studies of people aged 60 years and older found the prevalence of dry eyes ranged from 10.7% to 73.5% [9]. In those 80 years and older, a study by Schaumberg et al. reported dry eye prevalence to be around 7.7% [22]. Another study conducted in France found 21.9% of older adults, with a mean age of 80 years, had definite dry eyes [23]. The prevalence of dry eyes in our study appears to be higher than the aforementioned two studies. This could be because of differences in the definition of dry eyes and our sample being of older age and having higher rates of multimorbidity. Environmental factors, such as being confined to an indoor artificial climate, and dehydration may have also contributed [9]. 

Our study found that residents who used one or more medications listed as being known to cause, contribute to, or aggravate dry eyes were at 83% higher odds of having documented dry eyes or use of ocular lubricants compared to non-users. This remained significant after performing sensitivity analyses to restrict our definition of dry eyes to a documented diagnosis in the medical records. While it is possible that underlying comorbidities were responsible for dry eyes, with systemic medications acting as a proxy, we adjusted for a range of specific dry-eye related systemic diseases and CCI in our analyses. Possible mechanisms by which systemic medications may exert dry eye symptoms are complex [10]. Plausible mechanisms include their effect on meibomian glands and conjunctival goblet cells [24]. These structures can be affected by medication via alteration of neurological innervation; additionally, since these areas are highly vascularized tissues, some medications can access them and hence exhibit a direct effect (24). Muscarinic receptors are found on the corneal and conjunctival cells and thought to have a proliferative effect [25], and cholinergic activation of the G-protein coupled muscarinic receptor leads to tear secretion by the lacrimal gland [10,26]. Medications with anticholinergic properties such as antidepressants, antihistamines, and anti-Parkinson’s may thus affect tear production [10].

The most prevalent medication listed as being known to cause, contribute to, or aggravate dry eyes was aspirin. Previous research on the possible association between aspirin and dry eyes is inconsistent with some studies showing a link with dry eyes [27], and others showing users of aspirin were less likely to have dry eyes [28]. It has been suggested that aspirin and ibuprofen can be secreted in tears and therefore may play a role in tear instability [10]. This may lead to irritation or can increase tear evaporation [10]. While these medications rarely cause ocular problems at routinely prescribed doses, it is possible that ocular problems may become clinically significant at higher doses [29]. However, residents in our study predominately used low-dose aspirin for cardioprotection and sub-analyses identified no association between aspirin use and dry eyes or use of ocular lubricants. Other commonly used medications listed as being known to cause, contribute to, or aggravate dry eyes were propranolol and diazepam, possibly explained by decreased lacrimation [29].

While all residents with dry eyes were taking a medication suspected of causing dry eyes, this was largely driven by vitamin use. The association between vitamin use and dry eyes is inconsistent in the literature. Large epidemiological studies have found associations between multivitamin use and dry eyes [30,31], while others have found no significant effect. As vitamins have not been clearly defined in previous studies, we kept our definition of vitamins deliberately broad, encompassing all those listed under ATC code A11. This included not only multivitamin combinations, but also vitamin D preparations which accounted for the majority of vitamin use in our study population [32]. Vitamin D supplementation has been found to improve dry eye symptoms including tear quality and ocular surface conditions [33], particularly in those with low vitamin D levels and who have symptoms refractory to conventional treatment [34]. Further research should thus investigate the temporal association between vitamin use and dry eyes in longitudinal studies.

Increasing frailty levels were found to be associated with higher odds of having documented dry eyes or use of ocular lubricants. This likely reflects functional impairment and an increased risk for adverse health outcomes [35]. While the association between dry eyes and frailty has been understudied, a few previous studies have found an association between vision impairment and frailty [36,37]. For example, a longitudinal study of 2836 English community-dwellers aged ≥60 years found that non-frail older adults who experience poor vision had a two-fold increased risk of becoming prefrail or frail over four years of follow-up [37]. Dry eyes may be particularly important in frail older adults as, if left untreated, this may lead to visual complications in this vulnerable population [6,23].

In our study, residents with moderate to severe dementia severity were found to be less likely to have dry eyes or use ocular lubricants (OR = 0.47). This may be because people with dementia are less likely to report subjective symptoms of dry eye; hence, they are less likely to be diagnosed with dry eyes and in turn less likely to receive regular lubricating eye drops or ointments. A previous study reported that visual problems were underreported in residents with dementia, which could be attributed to under-recognition linked to residents with cognitive impairment not describing or expressing their symptoms in the same manner as residents without cognitive impairment [38]. This highlights the importance of ongoing ophthalmic and visual care by an appropriate eye care professional for this vulnerable population [2]. Despite these findings, frailty and dementia severity were not found to be significantly associated with dry eyes when limiting the definition to a recorded diagnosis in the medical record. 

Our study has some limitations. Firstly, our definition of dry eyes was not based on whether or not residents actually experienced dry eyes on the day of assessment. Second, people with dry eyes may not have had this documented in the medical records. Conversely, residents charted lubricating eye drops/ointments may not have been currently experiencing dry eyes. We attempted to overcome this limitation by performing a sub-analysis in those with a documented diagnosis in the medical records. Third, we did not investigate the dose and duration of medications known or suspected to cause, contribute to, or aggravate dry eyes into account. This could influence the findings as those who are on higher doses or have taken exposure medications for a longer duration may have higher risk of dry eyes. Additionally, as we did not know the date of initiation of exposure medications, it was not possible to assess causality using an incident–user design. Fourth, this study did not use any subjective or objective clinical tests; rather, it relied on the documentation of a dry eye diagnosis or the regular administration of ocular lubricants. As a result, we could not determine if all participants with dry eyes were true cases of dry eye disease. Additionally, we were not able to assess severity of dry eyes, either through direct clinical assessment or through frequency of eye drop administration. Fifth, we did not consider other factors that can influence dry eyes such as environment and genetics in our analysis. Finally, given the cross-sectional study design, it is not possible to determine causality.

To the best of our knowledge this is the first study to specifically investigate the prevalence of dry eyes in residents of ACSs. Our findings are largely hypothesis generating but could positively impact current practice in several ways. First, our findings highlight the need for larger epidemiological studies to investigate the prevalence of dry eyes in ACSs and assess the possibility of a dose-response relationship between medication use and dry eyes. Second, our findings suggest the possible need to consider dry eyes when prescribing medications that are known to cause, contribute to, or aggravate this condition. In addition, a thorough eye examination of all residents in ACSs should be performed by general medical practitioners, optometrists, or ophthalmologists to identify and manage dry eyes in at-risk residents. Our findings suggest that this should include an assessment of medications that are known or suspected to cause dry eyes.

## 5. Conclusions

This study found that 30% of ACS residents have dry eyes or use ocular lubricants. While medications and frailty status may increase the risk of dry eyes, dementia severity was inversely associated. Larger studies are needed to confirm the possible association between specific medications and dry eyes in the residential aged care setting. 

## Figures and Tables

**Table 1:** Baseline characteristics of participants with and without dry eyes.

Characteristic	Dry Eyes	No Dry Eyes	Total
Total	116 (30.3 %)	267 (69.7 %)	383 (100 %)
Age, mean (SD)	88.32 (6.3)	87.19 (6.1)	87.53 (6.2)
84 or younger	25 (21.6 %)	83 (31.1 %)	108 (28.2 %)
85–90	44 (37.9 %)	105 (39.3 %)	149 (38.9 %)
Older than 90	47 (40.5 %)	79 (29.6 %)	126 (32.9 %)
Female	91 (78.5 %)	206 (77.2 %)	297 (77.6 %)
Ophthalmic conditions	72 (62.1 %)	152 (56.9 %)	224 (58.5 %)
Glaucoma	16 (13.8 %)	32 (12.0 %)	48 (12.5 %)
Glaucoma eye drops use	14 (12.1 %)	28 (10.5 %)	42 (11 %)
Macular degeneration	16 (13.8 %)	43 (16.1 %)	59 (15.4 %)
Cataracts	45 (38.8 %)	91 (34.1 %)	136 (35.5 %)
IOL	18 (15.5 %)	24 (9.0 %)	43 (11.0 %)
Impaired vision	24 (20.7 %)	45 (16.9 %)	69 (18.02 %)
Other conditions	15 (12.9 %)	20 (7.5 %)	35 (9.1 %)
Dry eye-related systemic diseases, mean (SD)	2.07 (0.91)	2.00 (0.99)	2.02 (0.97)
Diabetes	30 (25.9 %)	59 (22.1 %)	89 (23.2 %)
Arthritis	97 (83.6 %)	214 (80.2 %)	311 (81.2 %)
Parkinson’s disease	4 (3.5 %)	15 (5.6 %)	19 (5.0 %)
Thyroid	18 (15.6 %)	52 (19.5 %)	70 (18.3 %)
Asthma	19 (16.4 %)	36 (13.5 %)	55 (14.4 %)
Osteoporosis / fracture	58 (50.00 %)	131 (49.1%)	189 (49.4 %)
Gout	14 (12.1 %)	27 (10.1 %)	41 (10.7 %)
CCI, mean (SD)	2.65 (1.62)	2.42 (1.84)	2.49 (1.78)
FRAIL-NH, mean (SD)	5.10 (4.4)	4.5 (3.9)	4.7 (4.05)
DSRS > 18	49 (43.0 %)	124 (46.6 %)	173 (45.5 %)
Dry Eye-Related Systemic Medications			
Suspected Medications ^a^			
0–1	21 (18.1 %)	70 (26.2 %)	91 (23.8 %)
2–3	62 (55.9 %)	140 (55.3 %)	202 (55.5 %)
4 or more	33 (29.7 %)	57 (22.5 %)	90 (24.7 %)
Known Medications ^b^			
0	58 (50.0 %)	170 (63.4 %)	228 (59.5 %)
1	51 (44.0 %)	86 (32.2 %)	137 (35.8 %)
2–3	7 (6.0 %)	11 (4.1 %)	18 (4.7 %)

SD, standard deviation; IOL, intraocular implant; CCI, Charlson comorbidity index; and DSRS, Dementia Severity Rating Scale; a. Suspected medications include those that have probable or possible relationship to dry eye symptoms; b. Known medications include those that have been determined to cause dry eye symptoms by withdrawal and rechallenge tests.

**Table 2:** Odds ratios and 95% confidence intervals for the association between systemic medications, dementia, and dry eyes.

Variable	Model 1 ^a^		Model 2 ^b^	
Suspected medications	OR (95% CI)	*p*-value	OR (95% CI)	*p*-value
Number of medications				
1 or more	1.28 (0.44, 3.67)	0.65	1.43 (0.47, 4.33)	0.53
0–1	1.00 (reference)		1.00 (reference)	
2–3	1.46 (0.82, 2.60)	0.20	1.45 (0.80, 2.62)	0.22
4 or more	1.97 (1.02, 3.81)	0.04	1.84 (0.93, 3.66)	0.08
Known medications	OR (95% CI)	*p*-value	OR (95% CI)	*p*-value
1 or more	1.78 (1.13, 2.80)	0.01	1.83 (1.15, 2.94)	0.01
0	1.00 (reference)		1.00 (reference)	
1	1.76 (1.11, 2.82)	0.02	1.90 (1.16, 3.08)	0.01
2–3	1.92 (0.70, 5.28)	0.21	1.43 (0.48, 4.25)	0.52
DSRS >18 ^c^	0.85 (0.54, 1.36)	0.47	0.47 (0.25, 0.88)	0.02
FRAIL-NH score ^c^	1.03 (0.98, 1.09)	0.25	1.11 (1.02, 1.19)	0.01

OR, odds ratio; CI, confidence interval; NSAIDs, non-steroidal anti-inflammatory drugs; and DSRS, Dementia Severity Rating Scale; ^a^. Adjusted for age and sex; ^b^. Adjusted for age, sex, ophthalmic conditions, dry eye-related systemic conditions, Charlson comorbidity index, DSRS, and FRAIL-NH; c. The DSRS and FRAIL-NH Model 2 was additionally adjusted for 1 or more known dry eye-related medications.

## Supplementary Materials

The following are available online at https://www.mdpi.com/1660-4601/17/15/5349/s1, Table S1: Medications suspected or known to cause, contribute or aggravate dry eyes, Table S2: Odds ratios and 95% confidence intervals for the association between systemic medications, dementia and dry eye disease diagnosis documented in medical record.
