# Supplementary material for: Dry Eyes, Ocular Lubricants, and Use of Systemic Medications Known or Suspected to Cause Dry Eyes in Residents of Aged Care Services
Source: Int J Environ Res Public Health. 2020 Jul 24;17(15):5349. doi: 10.3390/ijerph17155349 (PMC7432788; doi:10.3390/ijerph17155349)
Supplement: Supplementary file 1 [file ijerph-17-05349-s001.pdf]

Supplementary Materials: The following are available online at www.mdpi.com/xxx/s1, Table S1: Medications suspected or known to cause, contribute or aggravate dry eyes, Table S2: Odds ratios and 95% confidence intervals for the association between systemic medications, dementia and dry eye disease diagnosis documented in medical record.

Electronic Supplementary Material

Table S1. Medications suspected or known to cause, contribute or aggravate dry eyes *.

Drug	ATC Code	Known Dry Eye Effect
**Antipsychotics**		
ziprasidone	N05AE04	No
aripiprazole	N05AX12	No
chlorpromazine	N05AA01	No
clozapine	N05AH02	No
haloperidol	N05AD01	No
lithium carbonate	N05AN	No
olanzapine	N05AH03	No
quetiapine	N05AH04	No
risperidone	N05AX08	No
sulpiride	N05AL01	No
trifluoperazine	N05AB06	No
**Anxiolytics/Hypnotics and Sedatives**		
alprazolam	N05BA12	Yes
diazepam	N05BA01	Yes
lorazepam	N05BA06 OR N05BA56	Yes
zolpidem	N05CF02	No
zopiclone	N05CF01	No
**Antidepressants**		
agomelatine	N06AX22	No
amitriptyline	N06AA09	No
bupropion	N06AX12	No
clomipramine	N06AA04	No

citalopram	N06AB04	No
doxepin	N06AA12	No
duloxetine	N06AX21	No
fluoxetine	N06AB03	No
fluvoxamine	N06AB08	No
imipramine	N06AA02	No
mianserin	N06AX03	No
mirtazapine	N06AX11	No
nortriptyline	N06AA10	No
paroxetine	N06AB05	No
reboxetine	N06AX18	No
sertraline	N06AB06	No
venlafaxine	N06AX16	No
**Non-Steroidal Anti-Inflammatory Drugs (NSAIDs)**		
aspirin	N02BA01 OR N02AJ07 OR B01AC06	Yes
ibuprofen	M01AE01 OR M01AE51 OR N02AJ08	Yes
**Analgesics**		
morphine	N02AA01	Yes
buprenorphine	N02AE01 OR N07BC51	No
fentanyl	N02AB03	No
methadone	N07BC02	No
tapentadol	N02AX06	No
**Diuretics**		
hydrochlorothiazide	C03AA03 OR C03AX01 OR C03EA01 OR C03AB03 OR C09BX03	No
chlortalidone	C03BA04 OR C03BB04 OR C03EA06	No
indapamide	C03BA11 OR C09BX01 OR C10BX13	No
**Antihypertensives**		
clonidine	C02AC01	Yes

oxprenolol	C07AA02	Yes
propranolol	C07AA05	Yes
atenolol	C07AB03 OR C07FB03 OR C07CB03 OR C07CB53 OR C07BB03 OR C07DB01	No
carvedilol	C07AG02	No
labetalol	C07AG01	No
metoprolol	C07AB02	No
pindolol	C07AA03	No
prazosin	C02CA01	No
**Antiarrhythmics**		
disopyramide	C01BA03	No
**Antihistamines systemic**		
brompheniramine	R06AB01	Yes
cetirizine	R06AE07	Yes
chlorpheniramine	R06AB54	Yes
cyproheptadine	R06AX02	Yes
desloratadine	R06AX27	Yes
dexchlorpheniramine	R06AB02	Yes
diphenhydramine	R06AA02	Yes
doxylamine	R06AA09 OR R06AA59	Yes
fexofenadine	R06AX26	Yes
loratadine	R06AX13	Yes
triprolidine	R06AX07	Yes
azelastine	R06AX19	No
ketotifen	R06AX17	No
promethazine	R06AD02	No
pseudoephedrine	R01BA02 OR R01BA52	No
diphenhydramine	R06AA02 OR R06AA52	No
**Hormonal Replacement Therapy (HRT)**		

HRT	G03A OR G03C OR G03F OR G03XC	No
**Urologicals**		
finasteride	G04CB01	Yes
leuprorelin	L02AE02	Yes
tolterodine	G04BD07	Yes
alfuzosin	G04CA01	No
tamsulosin	G04CA02 OR G04CA52 OR G04CA53	No
terazosin	G04CA03	No
oxybutynin	G04BD04	No
propantheline	A03AB05	No
solifenacin	G04BD08	No
**Inhaled Medication**		
Ipratropium	R03BB01	Yes
tiotropium bromide	R03BB04 OR R03BB54	No
**Decongestants**		
azelastine	R01AC03	No
ketotifen	S01GX08	No
olopatadine	R01AC08	No
oxymetazoline	R01AA05 OR R01AB07	No
xylometazoline	R01AA07 OR R01AB06	No
phenylephrine	R01AA04 OR R01AB01	No
**Anti-Parkinson's**		
trihexyphenidyl	N04AA01	No
benzatropine	N04AC01	No
levodopa	N04BA01 OR N04BA02 OR N04BA03	No
orphenadrine	M03BC01	No
pramipexole	N04BC05	No
**Bisphosphonates**		
alendronic	M05BA04 OR M05BB03 OR M05BB05 OR M05BB06	No
pamidronic acid	M05BA03	No

risedronic acid	M05BA07 OR M05BB02 OR M05BB07 OR M05BB04	No
**Antineoplastic**		
Cetuximab	L01XC06	Yes
cyclophosphamide	L01AA01	Yes
erlotinib	L01XE03	Yes
gefitinib	L01XE02	Yes
panitumumab	L01XC08	Yes
vinblastine	L01CA01	Yes
verteporfin	S01LA01	Yes
busulfan	L01AB01	No
docetaxel	L01CD02	No
interferon	L03AB	No
methotrexate	L01BA01 OR L04AX03	No
mitomycin	L01DC03	No
**Antimuscarinic**		
atropine	A03BA01	Yes
scopolamine	A03BB01 OR A04AD01 OR N05CM05	Yes
**Anaesthetics**		
nitrous Oxide	N01AX13 OR N01AX63	Yes
ether	N01AA	Yes
**Antimalarial**		
chloroquine	P01BA01	Yes
hydrochloroquine	P01BA02	Yes
**Antileprosy**		
clofazimine	J04BA01	Yes
**Depressant**		
ethanol	V03AZ01	Yes
**Herbal and Vitamins**		
vitamins	A11	No
**Neurotoxin**		

botulinum	M03AX01	No

* See Gomes JAP, et al. (2017) TFOS DEWS II iatrogenic report. Ocular Surface 15:511-38 for more information.

Table S2. Odds ratios and 95 % confidence intervals for the association between systemic medications, dementia and dry eye disease diagnosis documented in medical record.

**Variable**	**Model 1** [a]		**Model 2** [b]	
Suspected medications	OR (95 %CI)	*p*-value	OR (95 %CI)	*p*-value
Number of medications				
1 or more	3.50 (0.45, 27.01)	0.23	2.89 (0.36, 22.9)	0.32
0-1	1.00 (reference)		1.00 (reference)	
2-3	2.00 (0.83, 4.80)	0.12	1.84 (0.75, 4.49)	0.18
4 or more	3.21 (1.25, 8.21)	0.01	2.84 (1.08, 7.42)	0.03
Known medications	OR (95 %CI)	*p*-value	OR (95 %CI)	*p*-value
1 or more	2.35 (1.28, 4.31)	<0.01	2.51 (1.33, 4.73)	<0.01
0	1.00 (reference)		1.00 (reference)	
1	2.50 (1.35, 4.63)	<0.01	2.70 (1.42, 5.15)	<0.01
2-3	1.25 (0.26, 5.94)	0.78	1.12 (0.22, 5.78)	0.89
DSRS >18[c]	0.91 (0.50, 1.65)	0.75	0.99 (0.43, 2.28)	0.98
FRAIL-NH Score[c]	0.99 (0.92, 1.06)	0.12	0.99 (0.90, 1.10)	0.89

OR, odds ratio; CI, confidence interval; NSAIDs, non-steroidal anti-inflammatory drugs; DSRS, Dementia Severity Rating Scale; a. adjusted for age and sex; b. adjusted for age, sex, ophthalmic conditions, dry eye-related systemic conditions, Charlson's comorbidity index, DSRS and FRAIL-NH; c. the DSRS and FRAIL-NH Model 2 was additionally adjusted for 1 or more known dry eye-related medications.
